# Balancing Local and Global Dependencies in EEG Emotion Recognition via Multi-Scale Hybrid Attention

**DOI:** 10.3390/s26196244

**Published:** 2026-10-01

**Authors:** Yiren Ding, Yangfan Wang, Rui Wang, Tingting Ji

**Affiliations:** 1 College of Marine Life Sciences, Ocean University of China, Qingdao 266100, China; dingyiren@stu.ouc.edu.cn (Y.D.);; 2 School of Physics and Electronic Information, Yantai University, Yantai 264005, China; 3 Faculty of Information Science and Engineering, Ocean University of China, Qingdao 266100, China

**Keywords:** DSFConformer, Dynamic Sparse Feature Fusion, EEG emotion recognition, multi-scale convolution, Lite Transformer

## Abstract

Electroencephalography (EEG)-based emotion recognition remains challenging due to the low signal-to-noise ratio, non-stationarity, strong inter-subject variability, and the distribution of affect-related information across multiple temporal resolutions. Although recent deep architectures have increasingly combined multi-scale local feature extraction with global dependency modeling, the adaptive integration of multi-scale representations before global encoding remains comparatively underexplored. To address this issue, we propose DSFConformer, an EEG emotion-recognition framework integrating multi-scale spatiotemporal convolution, Dynamic Sparse Feature Fusion (DSFF), and lightweight Transformer-based global modeling. The multi-scale encoder captures complementary local EEG dynamics at different temporal resolutions, while DSFF performs sample-adaptive feature reweighting and sparse suppression to reduce the influence of redundant or noise-sensitive responses. A Lite Transformer is subsequently employed to model global contextual dependencies with reduced computational cost. Experiments on the SEED dataset were conducted under complementary evaluation protocols. DSFConformer achieved an accuracy of 96.08% under chronological block-wise segment-level cross-validation. Under the stricter trial-wise protocol, it achieved 78.81% accuracy, statistically comparable to EEG-Conformer at 78.77%, with no significant paired difference. Ablation analyses further demonstrate that multi-scale convolution and DSFF provide the primary recognition-performance gains, whereas the Lite Transformer mainly improves computational efficiency while preserving comparable accuracy. Cross-session and leave-one-subject-out analyses further assess model robustness and generalization. Overall, the results indicate that adaptive sparse fusion of multi-scale EEG representations provides an effective approach for balancing local representation learning, noise-aware feature integration, and efficient global dependency modeling.

## 1. Introduction

### 1.1. Background and Challenges

Emotion recognition has become an important research topic in affective computing because of its broad applications in intelligent human–computer interaction, mental health monitoring, adaptive learning, and personalized services [1,2]. Among different physiological signals, electroencephalography (EEG) is particularly attractive because it provides an objective and relatively privacy-preserving measure of affective states [3,4,5]. However, reliable EEG-based emotion recognition remains challenging because EEG signals exhibit a low signal-to-noise ratio, strong non-stationarity, and substantial inter- and intra-subject variability [6,7,8,9].

Another important challenge is that emotion-related EEG information is distributed across multiple temporal resolutions. Short-term oscillatory variations may contain transient affective responses, whereas longer-range temporal relationships provide complementary contextual information [10,11]. Effective EEG representation learning therefore requires a model to preserve local inductive biases for short-range signal patterns while also capturing broader temporal and inter-channel dependencies.

### 1.2. Motivation and Research Gap

Early EEG emotion recognition methods mainly relied on handcrafted spectral, statistical, and time–frequency features, while later deep-learning approaches increasingly adopted convolutional neural networks for automatic representation learning [12,13,14,15]. Convolutional architectures are well suited to extracting local oscillatory and spatial patterns because of their strong local inductive bias. Multi-kernel and multi-scale designs further extend their temporal receptive fields and enable representations to be learned at different resolutions [16,17,18].

In parallel, Transformer- and graph-based architectures have been introduced to capture long-range contextual dependencies and structured relationships among EEG channels [11,19]. Recent methods have also begun to combine multi-scale local feature extraction with global dependency modeling. For example, multi-scale convolution has been integrated with recurrent or attention-based architectures, while MSDCGTNet combines a multi-scale dynamic CNN with a gated Transformer [18,20]. Therefore, the combination of multi-scale convolution and global attention modeling itself is no longer an unexplored direction.

A more specific challenge concerns how representations obtained at different temporal scales are integrated before global modeling. Existing approaches commonly rely on concatenation, feature recalibration, attention-based weighting, gating, or high-level feature aggregation [16,17,18,20]. Although these strategies can combine complementary information, they do not primarily target explicit sample-adaptive sparse suppression of unreliable or noise-sensitive responses across aligned multi-scale representations. This limitation is particularly relevant for EEG emotion recognition, where discriminative information is subtle, distributed, and easily affected by artifacts and subject-specific variability. Hence, the key motivation of this work is not simply to combine multi-scale local modeling with global dependency modeling, but to introduce a noise-aware and sample-adaptive mechanism for selectively integrating multi-scale local representations before efficient global encoding.

### 1.3. Proposed Framework and Contributions

**Figure 1:**
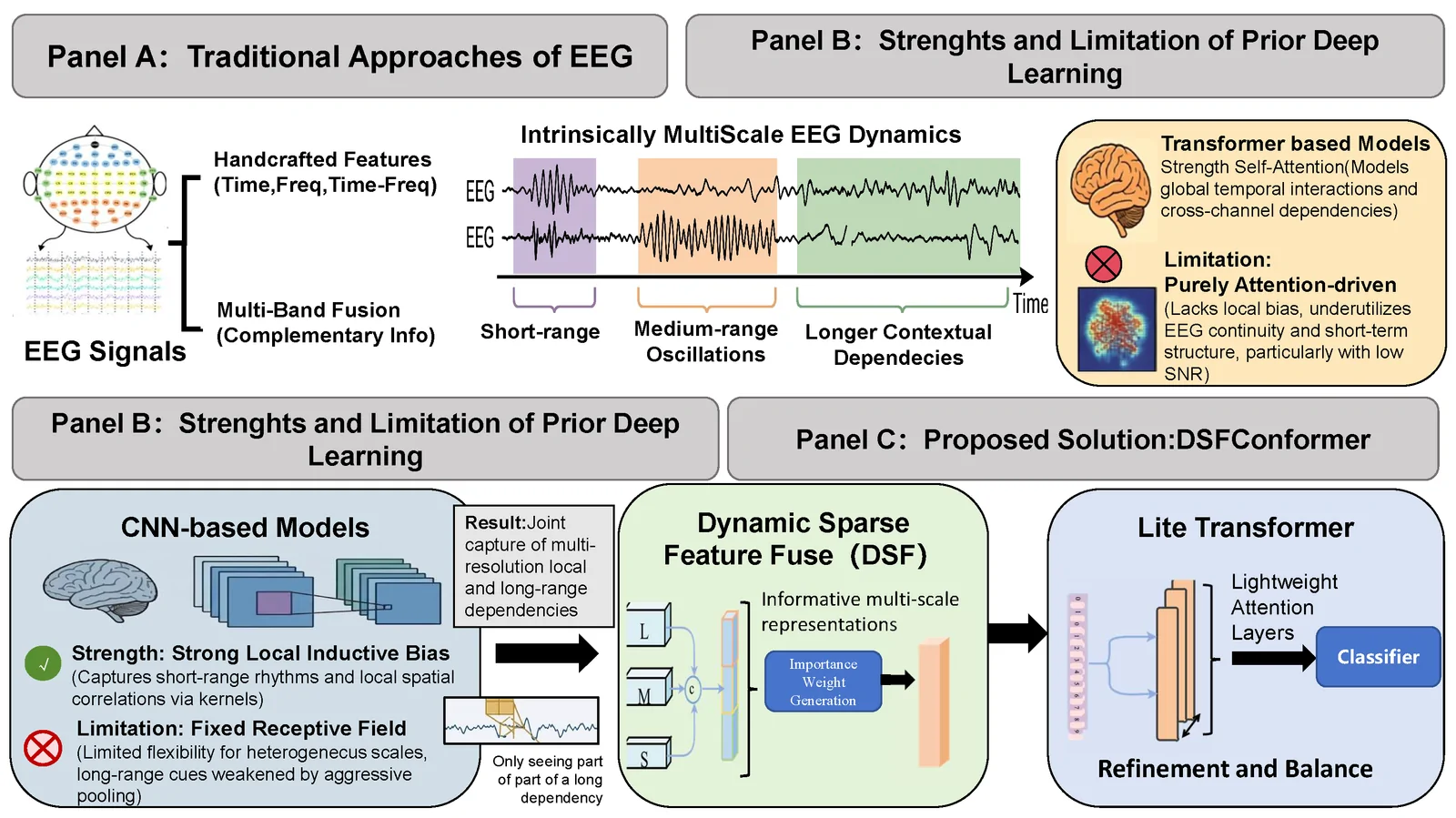
Motivation of the proposed framework. Multi-scale EEG representations and feature-fusion strategies have been explored in previous studies [17,18]. CNNs capture local oscillatory patterns, whereas Transformers model global dependencies; effective EEG emotion decoding requires multi-scale representations and principled fusion across resolutions.

Figure 1 illustrates the motivation of the proposed framework. To address the above challenges, we propose DSFConformer, a multi-scale convolutional Lite Transformer framework for EEG-based emotion recognition. DSFConformer follows a local-to-global representation learning pipeline consisting of multi-scale spatiotemporal encoding, Dynamic Sparse Feature Fusion (DSFF), and lightweight Transformer-based global modeling. The multi-scale encoder extracts complementary local EEG representations at different temporal resolutions, DSFF adaptively emphasizes informative responses while sparsely suppressing unreliable components, and the Lite Transformer subsequently captures global contextual dependencies with reduced computational cost.

The main contributions of this work are summarized as follows:

(1) A multi-scale spatiotemporal convolutional encoder is developed to capture complementary local EEG dynamics at short, medium, and long temporal resolutions. The parallel receptive fields provide a structured representation of heterogeneous temporal patterns while preserving the local inductive bias of convolution.
(2) A Dynamic Sparse Feature Fusion (DSFF) mechanism is introduced to perform sample-adaptive integration of aligned multi-scale representations. By dynamically emphasizing informative responses and sparsely suppressing redundant or noise-sensitive components, DSFF provides a noise-aware representation before global dependency modeling.
(3) A Lite Transformer is employed for efficient global dependency modeling after multi-scale feature fusion. Its role is primarily to reduce computational cost while maintaining effective global representation capability, rather than to introduce an additional structural accuracy gain.
(4) A comprehensive evaluation is conducted on the SEED dataset using complementary validation settings, including chronological block-wise segment-level cross-validation, stricter trial-wise cross-validation, statistical significance analysis, ablation studies, computational-efficiency analysis, cross-session evaluation, and leave-one-subject-out cross-subject testing. These experiments assess not only recognition performance but also robustness, efficiency, and generalization.

## 2. Related Work

### 2.1. Task-Related Emotion Recognition Studies

EEG-based emotion recognition has been extensively studied as an important task in affective computing [21,22]. Early studies mainly relied on handcrafted features extracted from time-domain, frequency-domain, or time–frequency representations [23,24]. In particular, differential entropy, band-specific spectral descriptors, and other statistical features were commonly combined with conventional classifiers to characterize emotion-related EEG activity. Although these approaches established the feasibility of EEG-based emotion recognition, their performance depended strongly on feature engineering and was often sensitive to inter-subject variability and non-stationary recording conditions [25,26].

With the development of deep learning, end-to-end neural architectures have gradually become a major direction for EEG emotion recognition [13,15,21]. CNN-based models enable automatic extraction of local temporal and spatial patterns from raw or minimally processed EEG signals, while recurrent, attention-based, graph-based, and Transformer-based architectures further extend the ability to model temporal dependencies, inter-channel relationships, and contextual information [11,18,19].

Recent studies have explored different strategies for improving EEG representation learning. Liu et al. [27] combined CNN-based feature extraction with a sparse autoencoder and a deep neural network to reduce feature redundancy and improve emotion classification. Yao et al. [28] proposed EEG ST-TCNN, in which parallel spatial and temporal Transformer encoders extract complementary representations that are subsequently aggregated by a CNN. Ju et al. [29] introduced a temporal-difference minimizing neural network that incorporates temporal continuity into a CNN–LSTM framework through distribution-level regularization. These studies demonstrate that EEG emotion recognition can benefit from feature compression, spatial–temporal dependency modeling, and temporal-prior constraints. Nevertheless, the low signal-to-noise ratio, strong non-stationarity, and substantial inter-subject variability of EEG signals remain important challenges [3,5,7].

### 2.2. Studies Related to Multi-Scale Local Modeling and Global Dependency Modeling

Emotion-related EEG patterns may occur over different temporal resolutions, motivating the development of multi-scale convolutional architectures. Su et al. [14] employed multi-scale 3D convolution to jointly characterize spatial and temporal EEG information. MultiT-S ConvNet [16] used multiple temporal kernels together with spatial convolution and feature recalibration, while TSception [17] introduced multi-scale temporal convolutions and asymmetric spatial filters to capture temporal dynamics and hemispheric characteristics. More recently, MSBiLSTM-Attention [18] combined multi-scale convolution with recurrent temporal modeling and attention-based feature integration. These studies demonstrate the effectiveness of multi-resolution local representations, but convolutional or recurrent modeling alone provides limited capability for directly capturing long-range dependencies across EEG sequences.

To strengthen global contextual modeling, Transformer- and graph-based architectures have increasingly been incorporated into EEG decoding. EEG-Conformer [11] combines convolutional feature extraction with Transformer-based self-attention, providing a representative local-to-global modeling framework. BGAGCN-MT [19] further integrates graph-based representation learning with multi-scale Transformer modeling to characterize structured relationships among EEG channels. Importantly, recent architectures have already begun to combine multi-scale local extraction with global attention modeling. For example, MSDCGTNet [20] integrates a multi-scale dynamic CNN with a gated Transformer and temporal convolutional modeling. Therefore, the combination of multi-scale convolution and Transformer-based global modeling itself should not be regarded as an unexplored problem.

The remaining distinction lies more specifically in how multi-scale local representations are integrated before global dependency modeling. Existing representative approaches employ strategies such as feature recalibration, high-level feature fusion, attention-based weighting, and concatenation or gating [16,17,18,20]. Although these mechanisms can combine complementary representations, they do not primarily focus on explicit sample-adaptive sparse suppression of unreliable or noise-sensitive responses across aligned temporal scales before global encoding. This issue is particularly relevant to EEG signals because discriminative affective information is distributed across temporal resolutions and can be easily contaminated by noise.

Table 1 summarizes representative EEG-based emotion recognition methods in terms of datasets, input representations, feature extraction, model optimization, evaluation protocols, and reported accuracy. Because these studies employ different datasets, input representations, segment lengths, and validation protocols, their reported accuracies should be interpreted within their respective experimental settings rather than as strictly direct performance comparisons.

As summarized in Table 1, current EEG emotion recognition architectures have progressively evolved from handcrafted-feature and convolution-based approaches toward multi-scale, recurrent, graph-based, and Transformer-based representation learning. In particular, several recent methods already combine multi-scale feature extraction with global dependency modeling, indicating that simply integrating multi-scale convolution with a Transformer is no longer a sufficient methodological distinction. However, the comparison also reveals that adaptive integration of multi-scale local representations remains less explicitly addressed. Existing methods generally rely on concatenation, attention-based reweighting, gating, or high-level feature aggregation, whereas explicit sample-specific sparse suppression of unreliable multi-scale responses prior to global modeling remains comparatively underexplored.

Accordingly, the research gap addressed in this work is not the absence of multi-scale or local–global modeling individually, but the lack of an integrated mechanism that jointly performs multi-scale local representation learning, sample-adaptive and noise-aware sparse feature fusion, and efficient global dependency modeling. Motivated by this gap, DSFConformer introduces Dynamic Sparse Feature Fusion (DSFF) between the multi-scale convolutional encoder and the Lite Transformer, allowing informative responses to be adaptively emphasized while redundant or noise-sensitive components are suppressed before global contextual modeling.

## 3. Methodology

### 3.1. Overview

Given an input EEG segment $X\in R^{C\times T}$, where *C* denotes the number of EEG channels and *T* denotes the number of temporal samples, the goal of EEG-based emotion recognition is to predict its emotion label y∈{1,…,K}, where *K* is the number of emotion categories. The proposed framework, termed DSFConformer, is designed to jointly model multi-scale local EEG dynamics and global contextual dependencies in a unified pipeline.

**Figure 2:**
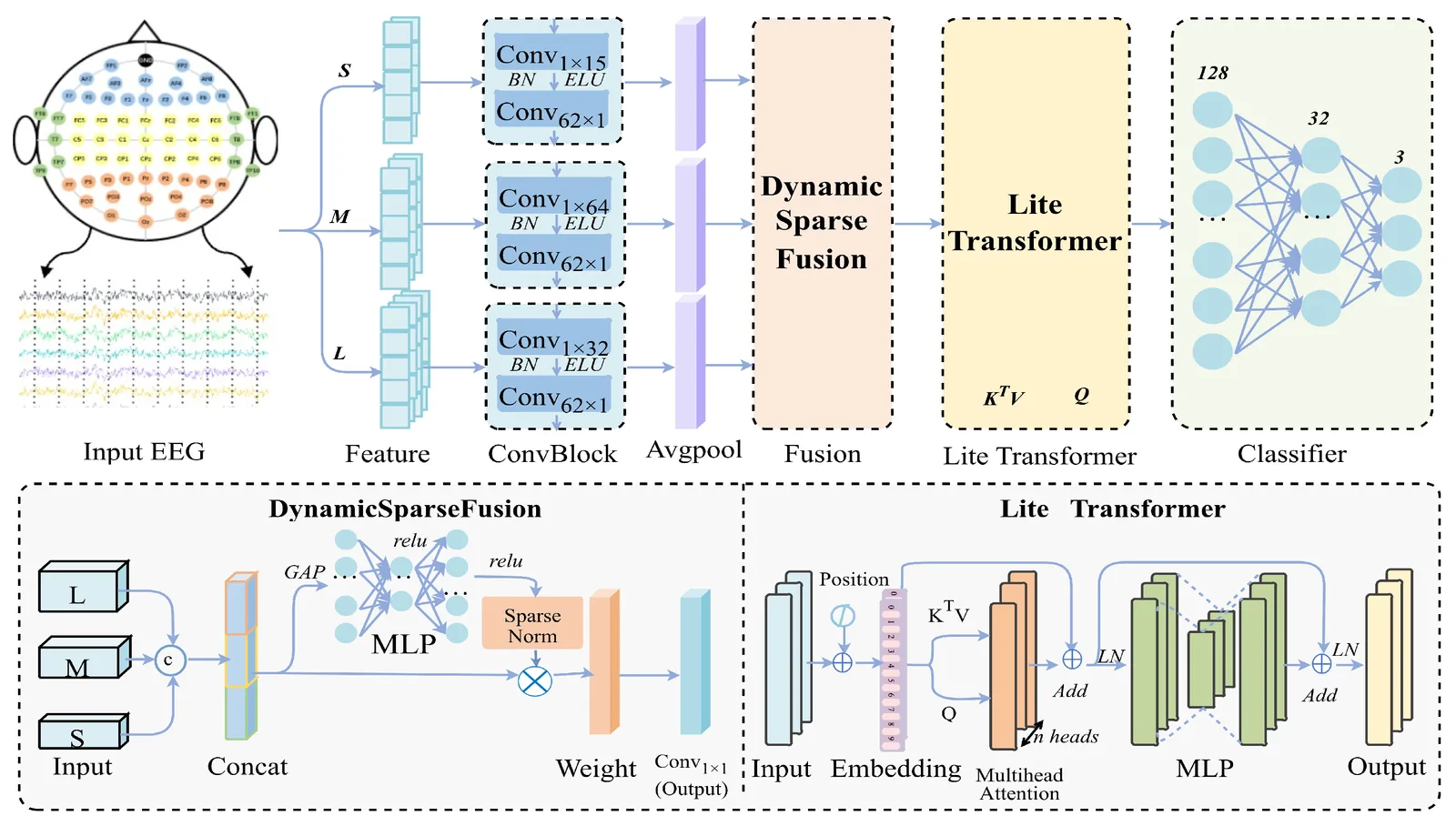
Overall architecture of the proposed DSFConformer for EEG-based emotion recognition, including dynamic multi-scale convolutional encoding, sparse feature fusion, Lite Transformer-based global dependency modeling, and final emotion classification.

As illustrated in Figure 2, DSFConformer consists of three main stages. First, a dynamic multi-scale spatiotemporal encoder is employed to extract local EEG representations at different temporal resolutions. Second, a Dynamic Sparse Feature Fusion (DSFF) module is introduced to adaptively integrate multi-scale features and suppress unreliable components. Finally, the fused representation is projected into a compact token sequence and fed into a Lite Transformer encoder for global dependency modeling and emotion classification.

Formally, the overall mapping of the proposed framework can be written as

(1) $$\hat{y}=C\left(L\left(T\left(F\left(E(X)\right)\right)\right)\right),$$

where E(·) denotes the dynamic multi-scale spatiotemporal encoder, F(·) denotes the DSFF module, T(·) denotes token projection, L(·) denotes the Lite Transformer encoder, and C(·) denotes the classifier. The proposed framework explicitly integrates multi-scale temporal modeling, noise-aware adaptive fusion, and efficient global dependency modeling, which together form the core design of DSFConformer for EEG emotion recognition.

### 3.2. Dynamic Multi-Scale Spatiotemporal Encoding

Emotion-related EEG signals contain discriminative information at different temporal scales. Short-term temporal variations may reflect transient affective responses, whereas relatively longer temporal patterns can provide complementary contextual information for emotion discrimination. To capture such heterogeneous temporal dynamics, we design a multi-scale spatiotemporal encoder with *M* parallel branches, where each branch is associated with a different temporal receptive field. Specifically, the short-, medium-, and long-scale branches are implemented with temporal convolutional kernels of different sizes, rather than being manually assigned to predefined EEG frequency bands. This design enables the model to learn local EEG representations from multiple temporal resolutions in parallel while preserving the local inductive bias of convolution.

Given an EEG segment $X\in R^{C\times T}$, where *C* denotes the number of EEG channels and *T* denotes the temporal length, the *m*-th branch first applies a temporal convolution with kernel size $k_{m}$, followed by a spatial convolution across EEG channels. The output of the *m*-th branch is formulated as

(2) $$Z_{m}=\phi \left(\mathrm{BN}\left({\mathrm{Conv}}_{s}\left({\mathrm{Conv}}_{t}^{\left(k_{m}\right)}\left(X\right)\right)\right)\right),$$

where ${\mathrm{Conv}}_{t}^{\left(k_{m}\right)}\left(\cdot \right)$ denotes temporal convolution with kernel size $k_{m}$, ${\mathrm{Conv}}_{s}\left(\cdot \right)$ denotes spatial convolution across EEG channels, BN(·) denotes batch normalization, and ϕ(·) denotes a nonlinear activation function.

In our implementation, three parallel branches are used to capture short-, medium-, and long-scale temporal dynamics, with temporal kernel sizes of 15, 31, and 63 samples, respectively. Given the 200 Hz sampling rate of the SEED signals, these kernels correspond to temporal receptive fields of approximately 75, 155, and 315 ms, with reciprocal time scales of about 13.3, 6.5, and 3.2 Hz. These scales progressively cover faster to slower EEG dynamics and broadly overlap with temporal characteristics associated with alpha/beta-, theta-, and delta-related activity, although the branches are not explicitly constrained to predefined EEG frequency bands. The odd-valued kernel sizes facilitate symmetric padding, while their progressively increasing scales provide a hierarchical expansion of the temporal receptive field. The corresponding convolutional configurations are (1,15), (1,31), and (1,63), with strides of (1,2), (1,2), and (1,4), and paddings of (0,7), (0,15), and (0,31), respectively. These values were selected as a practical multi-scale configuration for the SEED dataset rather than as universally optimal kernel sizes for all EEG emotion-recognition datasets.

To align feature lengths across branches, scale-aware temporal downsampling is applied:

(3) $${\tilde{Z}}_{m}={\mathrm{Pool}}_{m}\left(Z_{m}\right),\;m=1,2,\ldots ,M,$$

where ${\mathrm{Pool}}_{m}\left(\cdot \right)$ denotes branch-specific pooling or striding. After this operation, all branch outputs are transformed into a common temporal length $T^{\prime }$, yielding aligned multi-scale features:

(4) $${\tilde{Z}}_{m}\in R^{C_{m}\times T^{\prime }},\;m=1,2,\ldots ,M.$$

The proposed multi-scale encoder provides two main advantages. First, it extends the temporal receptive field in a structured manner, enabling the model to capture both fine-grained and coarse-grained EEG dynamics. Second, it preserves EEG-specific local inductive bias through convolution, which is beneficial under limited training data and low signal-to-noise recording conditions.

### 3.3. Dynamic Sparse Feature Fusion

**Figure 3:**
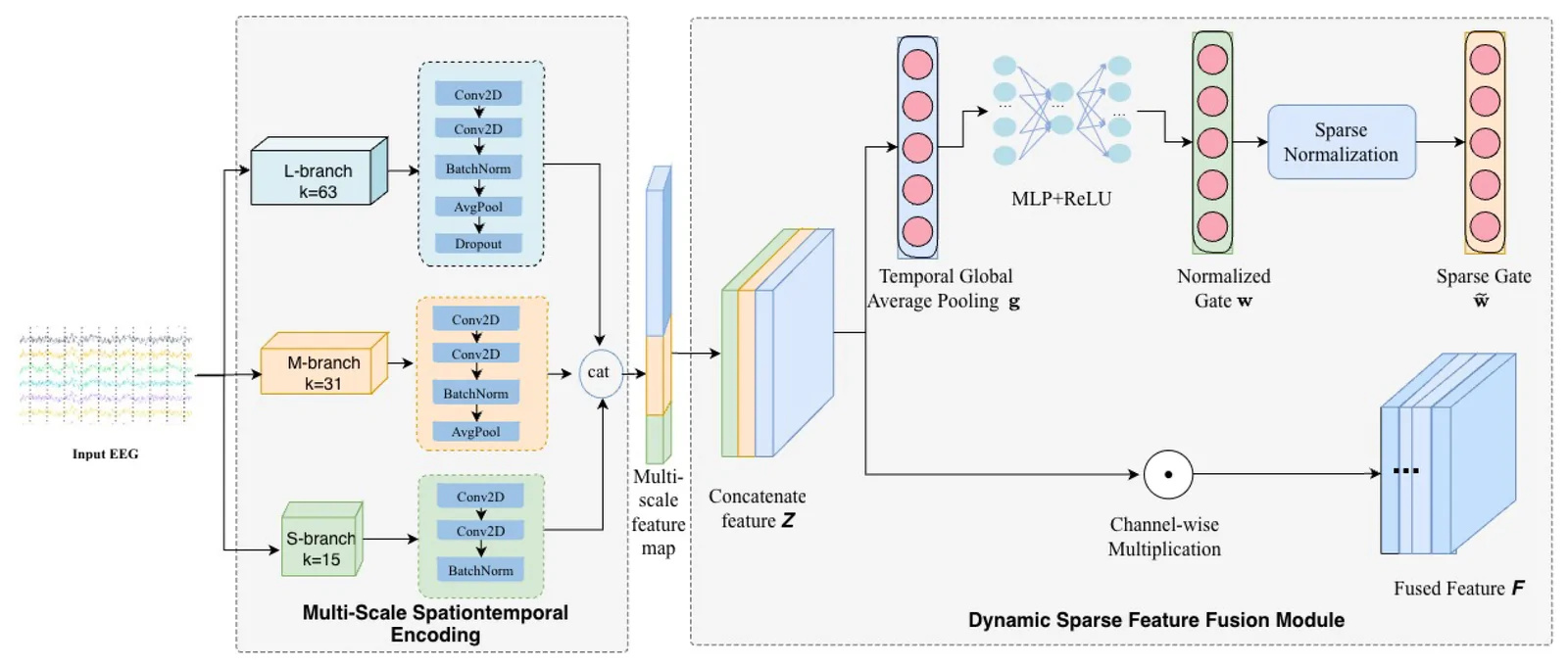
Architecture of the proposed Dynamic Sparse Feature Fusion (DSFF) module for adaptive integration of short-, medium-, and long-scale EEG features before Lite Transformer-based global dependency modeling.

Although multi-scale features are complementary, their reliability is not always consistent across samples. In EEG emotion recognition, some channels or temporal scales may be strongly affected by noise, artifacts, or subject-specific variability. Direct concatenation or averaging of all branch outputs may therefore introduce redundant or unreliable information into the subsequent global encoder. To address this issue, we propose a Dynamic Sparse Feature Fusion (DSFF) module, as shown in Figure 3. Different from static fusion strategies, DSFF performs sample-adaptive channel reweighting and sparse suppression, allowing the model to dynamically emphasize informative multi-scale responses while suppressing noise-sensitive components.

Given the aligned multi-scale features ${\left\{{\tilde{Z}}_{m}\right\}}_{m=1}^{M}$, we first concatenate them along the channel dimension:

(5) $$Z=\mathrm{Concat}\left({\tilde{Z}}_{1},{\tilde{Z}}_{2},\ldots ,{\tilde{Z}}_{M}\right),$$

where $Z\in R^{C^{\prime }\times T^{\prime }}$ and $C^{\prime }=\sum_{m=1}^{M}C_{m}$.

A global descriptor is then computed by temporal global average pooling:

(6) $$g=\frac{1}{T^{\prime }}\sum_{t=1}^{T^{\prime }}Z_{:,t},$$

where $g\in R^{C^{\prime }}$ summarizes the channel-wise responses of the current sample.

Based on g, a lightweight gating function is used to generate channel-wise importance weights:

(7) $$w=\mathrm{ReLU}\left(W_{2}\;\delta \left(W_{1}g\right)\right),$$

where $W_{1}$ and $W_{2}$ are learnable parameters, and δ(·) denotes a nonlinear activation function. The ReLU(·) operation is used to induce sparsity in the gate responses, encouraging uninformative or noise-sensitive channels to approach zero. In this way, DSFF explicitly suppresses unreliable components rather than merely assigning them small weights.

To stabilize the magnitude of the reweighted features, the gate vector is normalized as

(8) $$\tilde{w}=\frac{w}{\sum_{i=1}^{C^{\prime }}w_{i}+\varepsilon },$$

where ε is a small constant for numerical stability.

Finally, the fused representation is obtained by channel-wise reweighting:

(9) $$F=\tilde{w}⊙Z,$$

where ⊙ denotes channel-wise multiplication with broadcasting over the temporal dimension. The fused feature $F\in R^{C^{\prime }\times T^{\prime }}$ is then projected into token embeddings and passed to the Lite Transformer for global dependency modeling and final classification.

### 3.4. Lite Transformer-Based Global Modeling

After dynamic multi-scale local encoding and sparse feature fusion, the fused feature map still contains temporal dependencies and contextual relationships that cannot be fully captured by local convolution alone. To model such global interactions, we introduce a Lite Transformer module for EEG sequence modeling. Compared with a standard Transformer, the Lite Transformer is designed primarily to reduce computational cost while preserving comparable global dependency modeling capability. Rather than serving as an accuracy-enhancing component, its role is to improve the efficiency of global EEG sequence modeling without introducing a measurable loss in recognition performance.

Given the fused feature map $F\in R^{C^{\prime }\times T^{\prime }}$, we first project it into a compact embedding space and rearrange it into a sequence of token embeddings:

(10) $$S_{0}=\mathrm{Tokenize}\left(F\right)+E_{\mathrm{pos}},$$

where Tokenize(·) denotes the projection and rearrangement operation, and $E_{\mathrm{pos}}$ is a learnable positional embedding used to preserve temporal order information.

The token sequence is then processed by *N* stacked Lite Transformer blocks:

(11) $$S_{l}={\mathrm{LiteTrans}}_{l}\left(S_{l-1}\right),\;l=1,2,\ldots ,N,$$

where ${\mathrm{LiteTrans}}_{l}\left(\cdot \right)$ denotes the *l*-th Lite Transformer block.

Finally, the output token sequence is aggregated and fed into a classifier for emotion prediction:

(12) $$\hat{y}=\mathrm{Classifier}\left(\mathrm{Aggregate}(S_{N})\right),$$

where Aggregate(·) denotes token aggregation and $\hat{y}$ denotes the predicted emotion probability distribution.

## 4. Experiments and Analysis

In this section, we evaluate the proposed DSFConformer on the SEED dataset from multiple perspectives, including classification performance, component effectiveness, representation quality, and generalizability. We first describe the dataset, evaluation metrics, and implementation details. Then, we compare the proposed method with several state-of-the-art baselines and examine generalization abilities via cross-session and cross-subject protocols. Finally, we conduct ablation studies and visualization analyses.

### 4.1. Datasets

We evaluate the proposed method using the publicly available SJTU Emotion EEG Dataset (SEED) [23]. The present study is a secondary analysis of the existing SEED dataset and did not involve any new participant recruitment or EEG data collection. The number of participants and emotional stimuli was predetermined by the original SEED experimental protocol rather than selected by the authors of the present study.

According to the official SEED dataset documentation, EEG recordings are available from 15 Chinese participants (7 males and 8 females; mean age: 23.27 ± 2.37 years). The emotional stimuli were predetermined by the original SEED experimental design and consisted of 15 Chinese film clips, each lasting approximately 4 min, to elicit positive, neutral, and negative emotional states. The clips were selected to limit participant fatigue, ensure easy comprehension without additional explanation, and predominantly evoke a specific target emotion. Further individual-level demographic information and detailed inclusion/exclusion criteria are not reported on the official SEED dataset webpage and were therefore not assumed in the present secondary analysis.

Specifically, the dataset contains EEG recordings from 15 subjects, each of whom watched 15 film clips designed to elicit three emotional states: positive, negative, and neutral. Accordingly, no independent participant recruitment, stimulus selection, or sample-size determination was performed in this study. Following the standard preprocessing protocol, the raw EEG signals are band-pass filtered and segmented into non-overlapping short windows. Specifically, each EEG segment is represented as a matrix $X\in R^{C\times T}$, where C=62 channels and T=200 samples. The participant number, emotional stimuli, and trial protocol summarized in Table 2 follow the original SEED dataset design [23] and were not independently determined in the present study.

### 4.2. Model Evaluation Protocol, Implementation Details and Hyperparameter Settings

#### 4.2.1. Model Evaluation Protocol

To evaluate the proposed model under different levels of data separation and to account for the strong temporal correlation among adjacent EEG segments, we adopted two five-fold evaluation protocols: segment-wise chronological block-wise cross-validation and trial-wise cross-validation. Furthermore, regarding feature normalization, Z-score/in-channel normalization statistics (mean and variance) are computed strictly on the training partition and then applied to the validation/test partitions to prevent data snooping.

Following the evaluation protocol used by the end-to-end baseline [11], the continuous EEG recording from each stimulus trial was first divided into non-overlapping 1-s segments while preserving the original temporal order. For segment-wise five-fold cross-validation, the temporally ordered segments within each trial were further divided into five contiguous blocks. In the *k*-th fold, the *k*-th block from every trial was assigned to the validation set, while the remaining four blocks were used for training. This partitioning was performed independently within each trial before the resulting training and validation portions from different trials and sessions were aggregated. Therefore, the train–validation split was determined by chronological block position rather than by randomly mixing all EEG segments. After the partition was fixed, the training samples were shuffled only during mini-batch optimization, whereas the validation samples remained unshuffled. Thus, training-time shuffling affected only the presentation order of training samples and did not change the membership of the training and validation sets.

To further evaluate the model under a stricter setting, we conducted subject-wise trial-level five-fold cross-validation. For each subject, the 15 complete stimulus trials were divided into five non-overlapping folds, with three complete trials held out for validation and the remaining 12 used for training in each fold. The split was performed at the trial level before segment-level aggregation, ensuring that all 1-s segments from the same trial were assigned exclusively to either the training or validation set. This protocol was applied independently to each subject to assess within-subject generalization to unseen trials while controlling for inter-subject variability. A pooled all-subject trial-wise setting was not used because it would jointly introduce trial-level and subject-related variability, making the interpretation of unseen-trial generalization less specific; cross-subject generalization was instead evaluated separately using the leave-one-subject-out (LOSO) protocol described in Section 4.5.

#### 4.2.2. Implementation Details and Hyperparameter Settings

The proposed DSFConformer was implemented in Python 3.13.7 (Python Software Foundation, Beaverton, OR, USA) using PyTorch 2.9.1 (PyTorch Foundation, Linux Foundation, Wilmington, DE, USA) and trained on an NVIDIA GeForce GTX 1080 Ti GPU (NVIDIA Corporation, Santa Clara, CA, USA). Numerical computation and data processing were performed using NumPy 2.2.6 and pandas 2.3.3, respectively. Statistical analyses were conducted using SciPy 1.16.2. Figures were generated using Matplotlib 3.10.7, and EEG topographic maps were produced using MNE-Python 1.10.2. Hyperparameters are detailed in Table 3.

### 4.3. Comparison with State-of-the-Art Methods

#### 4.3.1. Comparison Under the Standard Protocol

We compared DSFConformer with several representative methods on the SEED dataset (Table 4). To ensure a fair comparison under consistent evaluation protocols, we reproduced EEG-Conformer and compared it under both segment-wise and trial-wise settings.

#### 4.3.2. Comparison Under the Trial-Wise Protocol

Table 5 and Table 6 report the subject-specific performance and statistical comparison under the trial-wise evaluation protocol.

As shown in Table 5, the trial-wise results show nearly identical group-average performance between DSFConformer and EEG-Conformer (78.81% vs. 78.77%). No statistically significant difference was observed according to either the paired *t*-test (p=0.984) or the Wilcoxon signed-rank test (p=0.847). The 95% confidence interval of the mean difference included zero ([−3.99, 4.06]), and the corresponding effect size was negligible (Cohen’s $d_{z}=0.005$). These results indicate that DSFConformer maintains performance comparable to EEG-Conformer under the stricter trial-wise evaluation protocol.

#### 4.3.3. Computational Efficiency Comparison

We conducted a controlled layer-wise comparison by isolating a single Transformer encoder layer from the proposed network and EEG-Conformer under standardized input tokens (sequence length N=25, embedding dimension D=80). Under the same input setting, the Lite attention module achieves lower parameter count, FLOPs, and inference latency than standard attention. These results support the role of the Lite Transformer as an efficiency-oriented component, intended to reduce computational cost rather than to serve as the primary source of recognition-performance improvement.

### 4.4. Ablation Studies and Analysis

To systematically analyze the individual contributions of multi-scale convolution, the DSFF module, and the Lite Transformer, we conducted ablation experiments as outlined in Table 7.

Compared with standard attention under the matched input setting, the Lite attention module achieves lower computational cost and inference latency while preserving comparable recognition performance. These results demonstrate that the proposed lightweight attention design provides an effective accuracy–efficiency trade-off for global dependency modeling.

**Figure 4:**
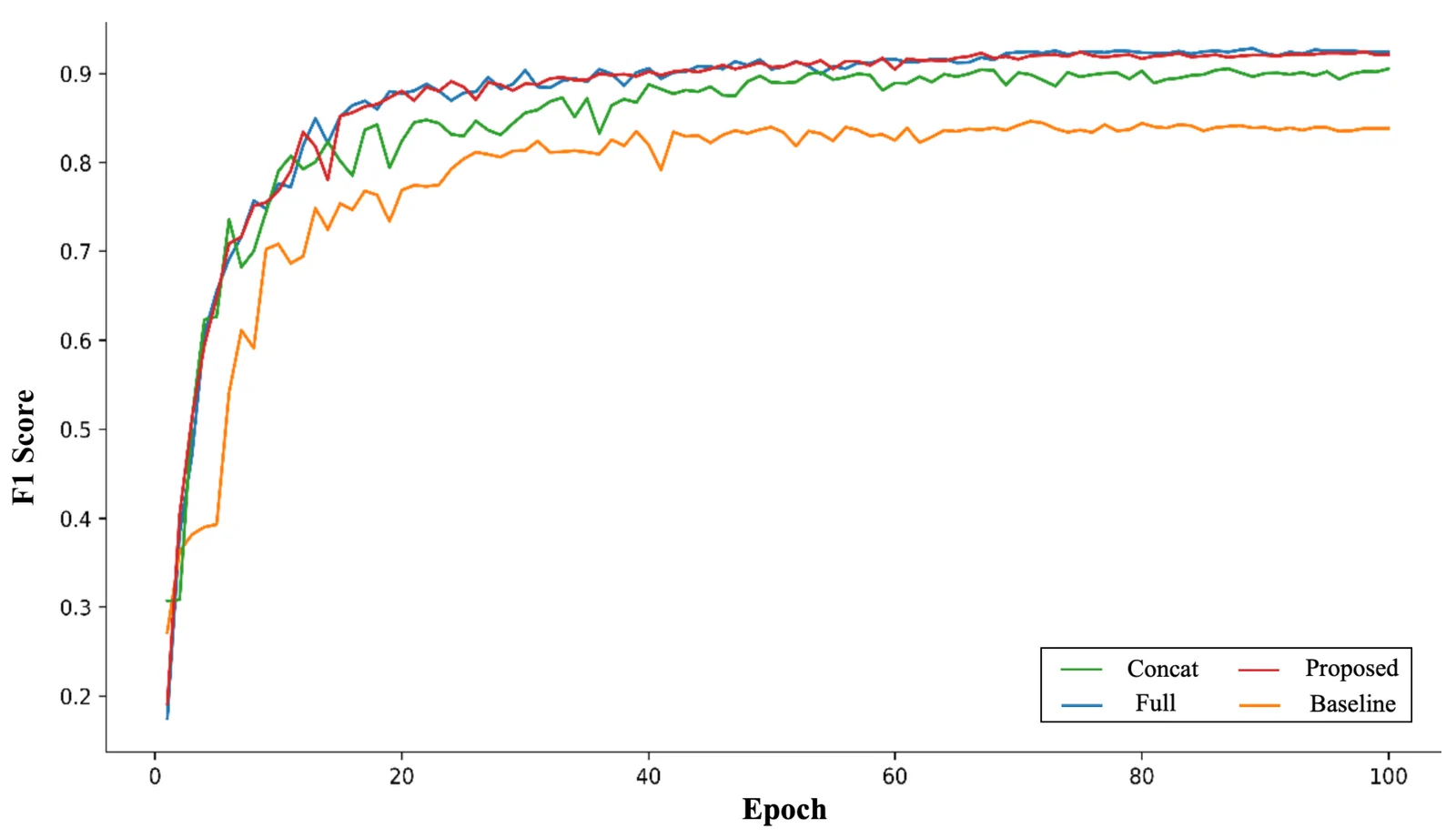
Comparison of F1-score for different ablation variants on the SEED dataset, showing the effects of multi-scale convolution, DSFF, and Lite Transformer-based global modeling.

As shown in Figure 4, the introduction of multi-scale convolution leads to a clear improvement over the single-scale baseline, indicating that EEG emotion-related patterns benefit from being modeled at multiple temporal resolutions. Further incorporating DSFF provides an additional improvement, suggesting that adaptive reweighting is beneficial beyond simply extracting multi-scale features. In contrast, replacing the full Transformer with the Lite Transformer produces only a minor change in recognition performance. This trend is consistent with the intended role of the Lite Transformer as an efficiency-oriented global modeling component rather than an accuracy-enhancing module.

As shown in Table 8, paired *t*-tests across 15 subjects (df=14) with Bonferroni correction (α=0.0167) confirm that multi-scale convolution and DSFF yield statistically significant performance improvements. In contrast, replacing the full Transformer in M2 with the Lite Transformer changes the mean accuracy by only +0.03%, with no statistically significant difference (*p* = 0.8388, 95% CI: [−0.24, +0.30]. Cohen’s dz = 0.053). Therefore, the Lite Transformer should not be interpreted as an accuracy-enhancing component. Its primary contribution is to preserve recognition performance while reducing computational complexity and improving execution efficiency, as further supported by the computational comparison in Table 9.

### 4.5. Model Generalization Analysis

To evaluate model robustness across non-stationary EEG sessions and unseen subjects, we incorporated the CORAL domain alignment loss [36].

**Figure 5:**
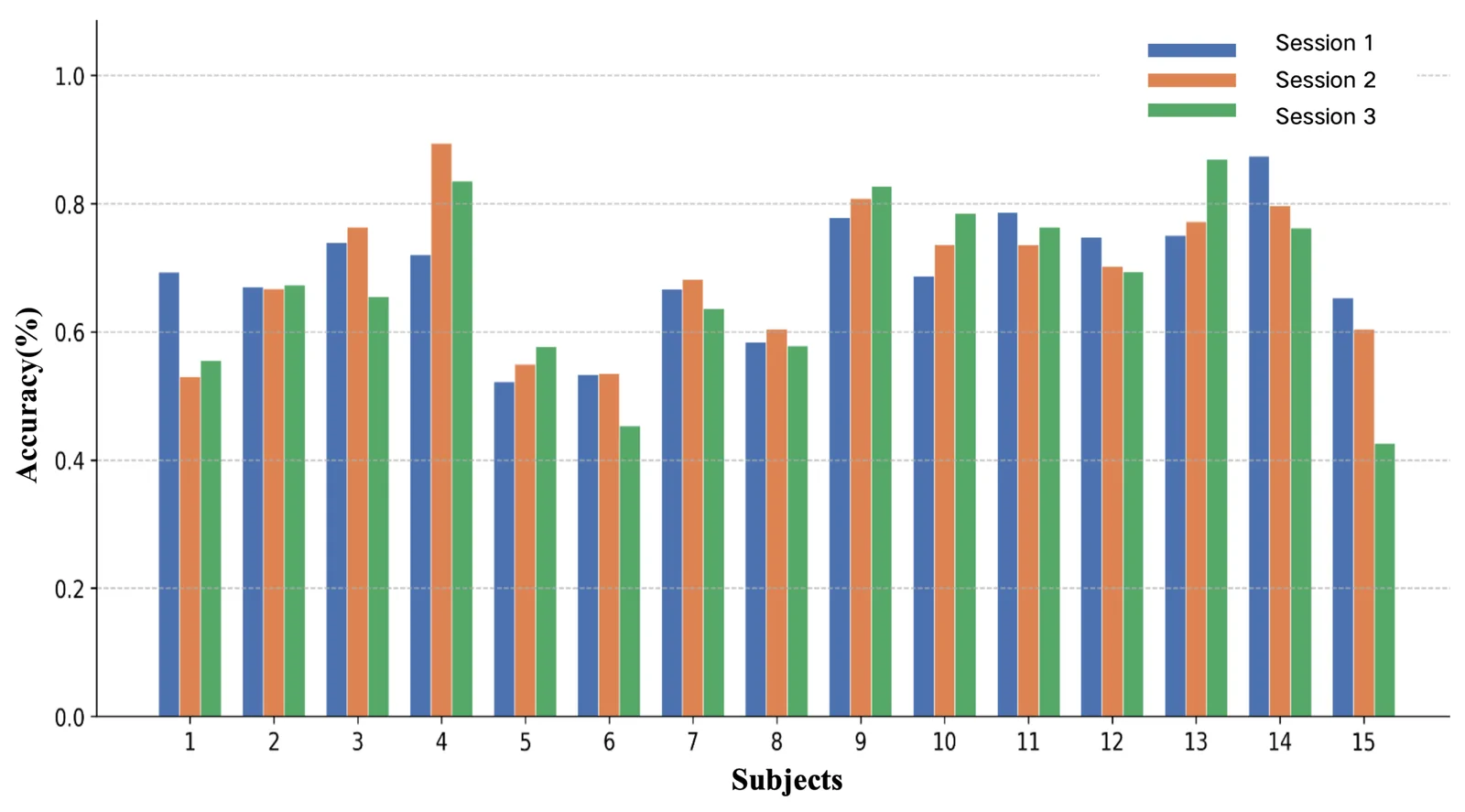
Cross-session emotion recognition performance of DSFConformer across 15 subjects and three independent experimental sessions.

As depicted in Figure 5, across the three sessions, the overall macro-level performance exhibits an average accuracy drop of only 2.1% between the first and third sessions, showing stable cross-session adaptation.

**Figure 6:**
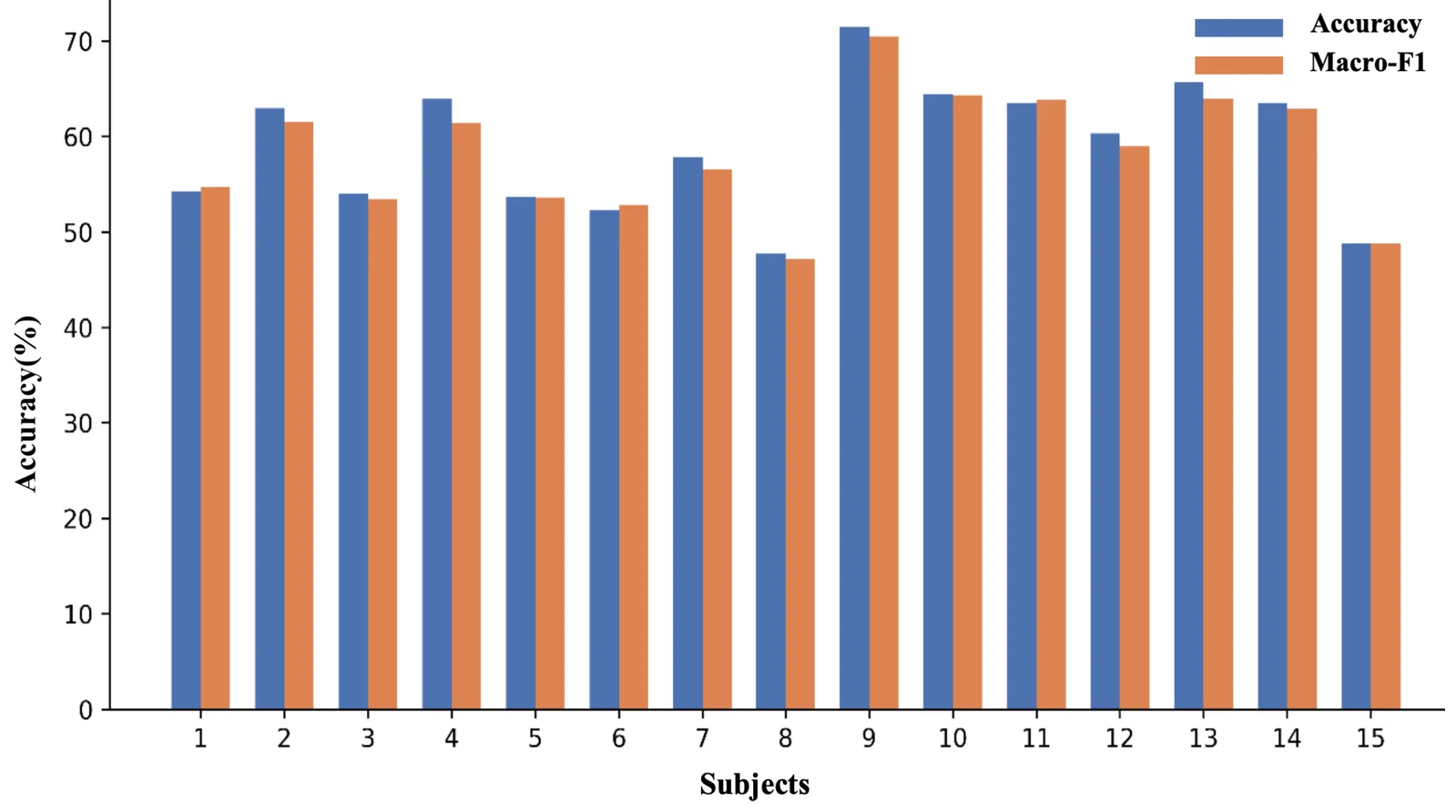
Cross-subject emotion recognition performance of DSFConformer across 15 subjects under the leave-one-subject-out (LOSO) protocol.

Under the Leave-One-Subject-Out (LOSO) cross-subject setting (Figure 6), when performing cross-subject adaptation via CORAL, unlabeled target-subject samples are used strictly for covariance alignment in an unsupervised transductive domain adaptation setting, without accessing any target emotional labels. Evaluated under this protocol, DSFConformer achieves an average accuracy of 58.96% and an average Macro-F1 score of 58.30%, surpassing the random-chance level of 33.3% in three-class affective classification.

Compared with the within-subject trial-wise evaluation, the lower LOSO performance reflects the substantially greater difficulty of generalizing to an entirely unseen participant. Under this setting, the model must cope not only with unseen EEG trials but also with strong inter-subject differences in signal distribution and affective response patterns. The above-chance performance therefore indicates that DSFConformer captures emotion-related representations that are partially transferable across subjects, while the remaining performance gap highlights cross-subject variability as an important challenge for subject-independent EEG emotion recognition.

### 4.6. Feature Representation Analysis

We analyzed the Transformer input features using Singular Value Entropy (SVE) [37,38] and PCA-based cumulative explained variance.

**Figure 7:**
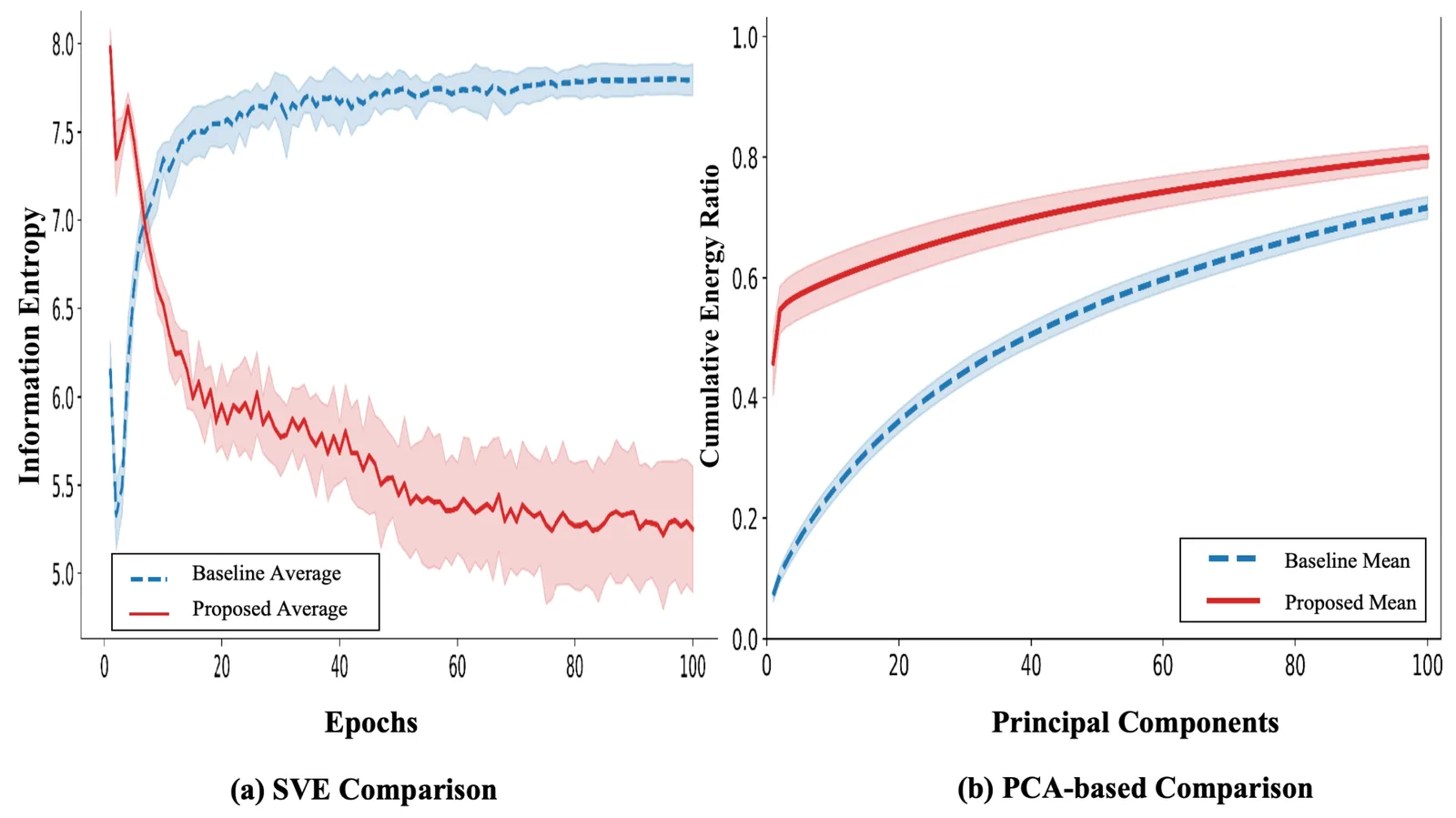
Feature representation analysis of Transformer input features: (**a**) SVE comparison during training; (**b**) PCA cumulative explained energy ratio. The curves represent the mean values across five cross-validation folds, and the shaded areas indicate ±1 standard deviation.

As shown in Figure 7, DSFConformer obtains a lower mean SVE (5.25 vs. 7.79) and requires only 10 principal components to capture 59.7% of the total energy, whereas the baseline requires 61 principal components to reach a comparable cumulative energy ratio. These results indicate that the Transformer input features learned by DSFConformer are concentrated within a lower-dimensional subspace and exhibit reduced representational redundancy. Importantly, lower SVE alone should not be interpreted as evidence of superior representation quality. However, when considered together with the stronger PCA energy concentration and the maintained recognition performance, the results suggest that DSFConformer organizes discriminative EEG information into a more compact feature representation rather than merely increasing feature dimensionality.

### 4.7. Visualization

**Figure 8:**
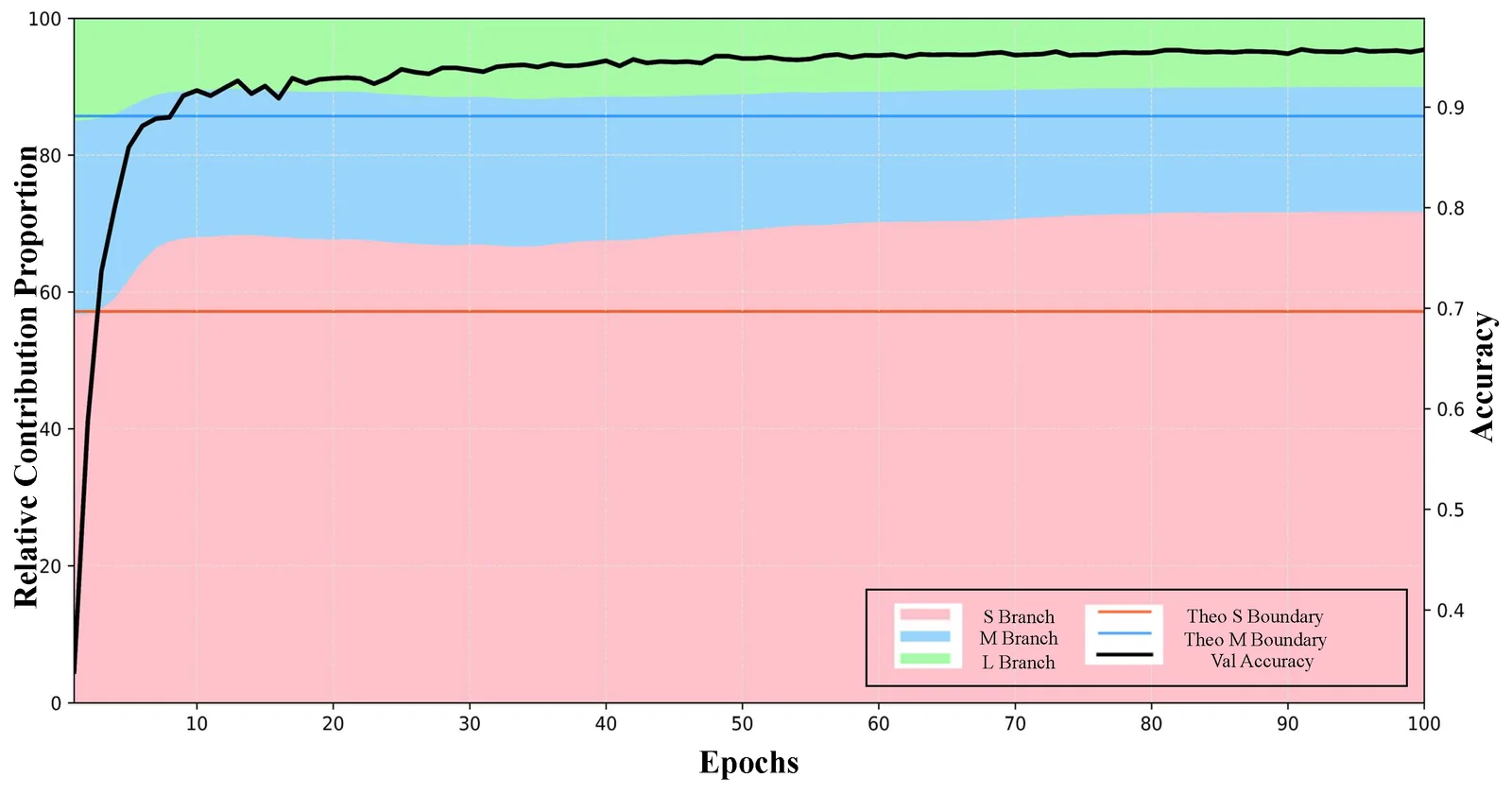
Evolution of DSFF-generated scale-wise feature contributions and validation accuracy during training.

As illustrated in Figure 8, the relative contributions of the three temporal branches evolve dynamically during training rather than remaining fixed. At the epoch corresponding to peak validation accuracy, the S-, M-, and L-branch contributions are 71.73%, 18.26%, and 10.01%, respectively, indicating that short-scale temporal representations provide the dominant contribution under the present SEED setting. Nevertheless, the persistent non-zero contributions of the medium- and long-scale branches suggest that broader temporal contexts remain complementary to short-scale dynamics. The evolving contribution ratios are consistent with the design motivation of DSFF, which adaptively redistributes feature importance across temporal scales instead of relying on a fixed fusion strategy.

**Figure 9:**
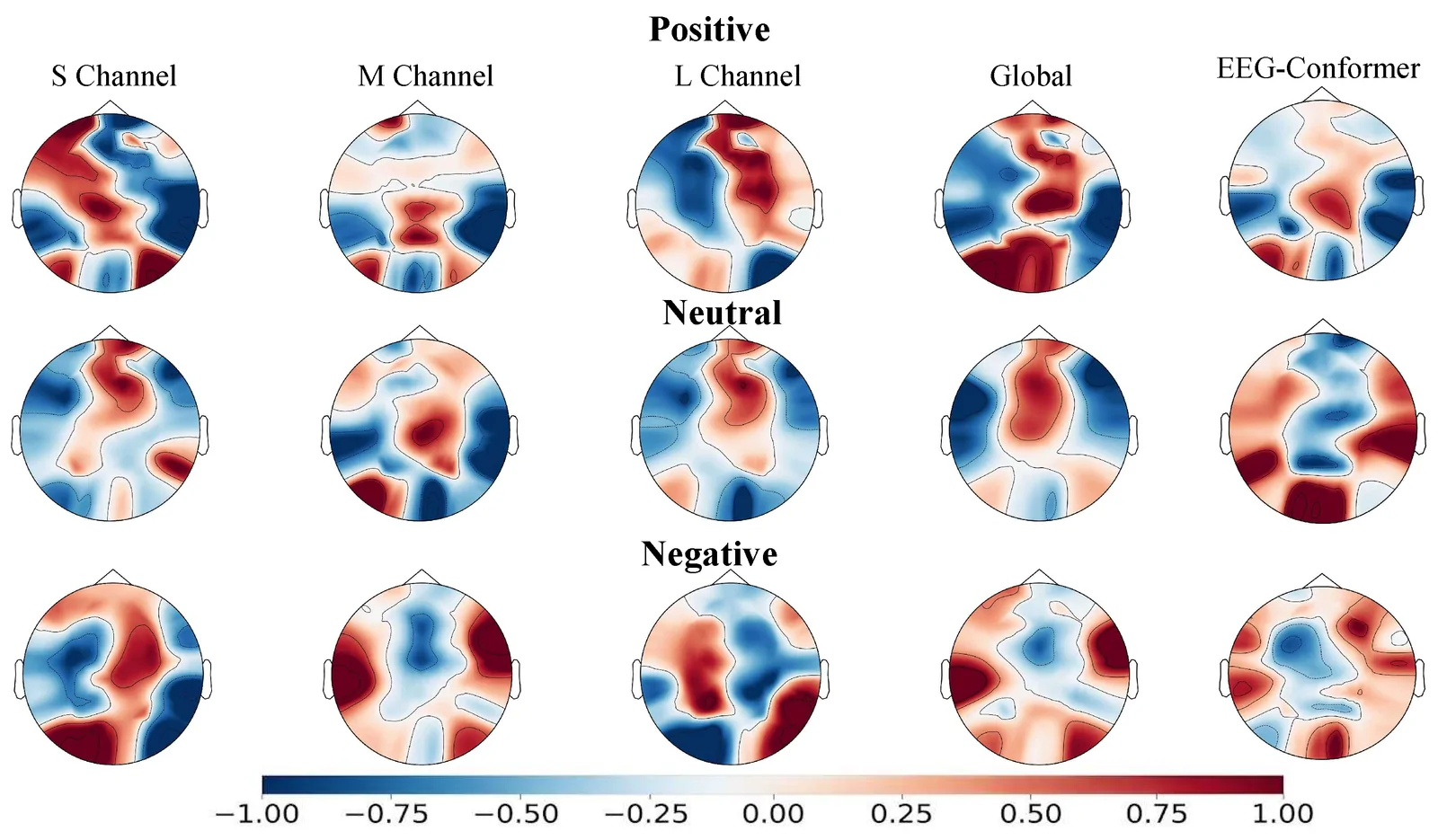
Topographic visualization of electrode-level saliency patterns for a representative subject (Subject 14) across positive, neutral, and negative emotional states. The first four columns show the short-scale, medium-scale, long-scale, and fused global representations of DSFConformer, respectively, while the fifth column shows the global representation of EEG-Conformer for qualitative comparison.

Figure 9 visualizes electrode-level saliency patterns for a representative subject (Subject 14) across the three emotional states. The S-, M-, and L-scale representations exhibit visibly different spatial distributions, suggesting that the parallel temporal branches capture complementary rather than redundant spatiotemporal information. The fused global representation does not simply reproduce any individual branch, but instead reflects the integration of salient patterns distributed across multiple temporal scales. Compared with EEG-Conformer, the DSFConformer global maps show partially overlapping but non-identical spatial organizations. Similarities under the positive and negative conditions suggest that both models capture several common affect-related spatial characteristics, whereas more noticeable differences are observed under the neutral condition. These observations suggest that multi-scale extraction and adaptive fusion modify the representation subsequently used for global dependency modeling. The observed frontal and temporal saliency is broadly consistent with previous affective EEG findings [23]; however, these maps represent model-level electrode saliency and should not be interpreted as direct evidence of cortical activation or causal neurophysiological mechanisms.

### 4.8. Practical Applications

Although the present study focuses on methodological evaluation rather than deployment in real-time affective systems, the proposed DSFConformer may provide value for offline EEG-based emotion analysis. In experimental affective neuroscience and human–computer interaction studies, EEG recordings are often analyzed after data acquisition to characterize emotional responses across trials or sessions. In such settings, the multi-scale convolutional encoder and DSFF module can provide a structured representation of affect-related EEG dynamics across different temporal resolutions, while the Lite Transformer supports subsequent global contextual modeling. Therefore, DSFConformer may serve as an analysis framework for retrospective or session-level characterization of EEG emotional states rather than as a dedicated real-time recognition system.

The framework may also support exploratory analysis of learned EEG representations. The multi-scale branches and fused representations provide a means to examine how information extracted at different temporal resolutions contributes to the final emotion-related representation. In particular, the electrode-level saliency maps show that the short-, medium-, and long-scale branches can emphasize different spatial patterns, while the fused representation integrates salient responses across scales. Such analyses may help researchers investigate the internal representation behavior of EEG emotion-recognition models and evaluate whether different temporal resolutions contribute complementary information. However, these visualization results should be interpreted as model-level explanations rather than direct evidence of cortical activation or causal neurophysiological mechanisms.

### 4.9. Limitations and Future Work

Several limitations of the present study should be acknowledged. First, cross-subject generalization remains challenging. Under the leave-one-subject-out evaluation, DSFConformer achieved an average accuracy of 58.96% and a Macro-F1 score of 58.30%. Although these results remain above the 33.3% random-chance level for three-class classification, they are substantially lower than the within-subject results, indicating that inter-subject variability continues to limit subject-independent EEG emotion recognition.

Second, the current evaluation is restricted to the SEED dataset, which contains EEG recordings from only 15 Chinese participants with a relatively narrow demographic distribution [23]. Therefore, the present results do not establish generalization to larger or more demographically diverse populations, different acquisition systems, or different electrode configurations. Validation on additional public datasets and larger heterogeneous cohorts will be necessary to determine whether the proposed multi-scale fusion strategy remains effective under broader recording and population conditions.

Third, the experimental paradigm and emotion-label space are relatively limited. SEED uses movie clips to induce three categorical emotional states—positive, neutral, and negative—which do not fully represent the complexity of naturalistic, mixed, or continuously varying affective states [23]. Consequently, the effectiveness of DSFConformer under other emotion-elicitation paradigms or finer-grained affective representations remains to be established.

Future work will therefore focus on three directions. First, stronger subject-invariant representation learning and domain-generalization strategies will be investigated to improve cross-subject robustness. Second, the proposed framework will be evaluated across additional EEG emotion datasets and larger, more demographically diverse populations to assess cross-dataset and population-level generalizability. Third, future studies will consider alternative emotion-elicitation paradigms and richer label spaces, including finer categorical emotion classes and continuous affective dimensions such as valence and arousal.

## 5. Conclusions

In this study, we proposed DSFConformer, an EEG emotion-recognition framework that integrates multi-scale spatiotemporal convolution, Dynamic Sparse Feature Fusion (DSFF), and lightweight Transformer-based global dependency modeling. Rather than treating the combination of convolution and Transformer modeling itself as the primary contribution, the proposed framework focuses on the adaptive integration of aligned multi-scale local representations before global encoding. DSFF performs sample-dependent feature reweighting and sparse suppression to emphasize informative responses while reducing the influence of redundant or noise-sensitive components.

Experimental results on the SEED dataset demonstrate that DSFConformer achieves competitive recognition performance under multiple evaluation settings. Under the chronological block-wise segment-level protocol, the proposed method achieved an accuracy of 96.08%. Under the stricter trial-wise protocol, DSFConformer achieved an average accuracy of 78.81%, which was statistically comparable to EEG-Conformer at 78.77%, with no significant paired difference. Ablation analysis further showed that multi-scale convolution and DSFF provide the primary performance gains, whereas replacing the full Transformer with the Lite Transformer causes no statistically significant change in recognition accuracy. These results support the intended role of the Lite Transformer as an efficiency-oriented global modeling component rather than an independent accuracy-enhancing module.

The generalization analyses also reveal remaining challenges. Although DSFConformer achieved above-chance performance under the LOSO cross-subject setting, the corresponding accuracy of 58.96% and Macro-F1 score of 58.30% remain substantially below the within-subject results, highlighting the difficulty of subject-independent EEG emotion recognition. In addition, the current study is limited to the SEED dataset, a relatively small and demographically restricted participant cohort, and a movie-induced three-class emotion paradigm. These factors constrain conclusions regarding broader population, dataset, and paradigm generalizability.

Overall, the results suggest that adaptive sparse fusion of multi-scale local EEG representations is a useful direction for balancing representation richness, robustness, and global contextual modeling. Future work will focus on improving subject-independent generalization, validating the framework across additional EEG datasets and more diverse populations, and extending the evaluation to alternative emotion-elicitation paradigms and richer affective label spaces.

## Figures and Tables

**Table 1:** Comparison of representative EEG-based emotion recognition methods.

Method	Dataset	Input	Feature Extractor	Optimization	Evaluation	Accuracy (%)
CNN-SAE-DNN [27]	SEED; DEAP	PCC features	CNN + SAE	SAE compression	Train/test split	SEED 96.77
Multiscale 3D-CNN [14]	DEAP; SEED-IV	EEG topology	Multi-scale 3D CNN	Multi-scale kernels	10-fold CV	95.67/89.55
MultiT-S ConvNet [16]	SEED; DEAP	EEG segments	Multi-kernel CNN	SE recalibration	SD/SI	SEED 86.0/54.6
TSception [17]	DEAP; MAHNOB-HCI	Raw EEG	Temporal-spatial CNN	High-level fusion	Trial-wise 10-fold	DEAP 61.57/59.14
MSBiLSTM-Attention [18]	SEED; SEED-IV	Raw EEG	Multi-scale CNN + BiLSTM	Attention fusion	10-fold CV	99.49/99.70
BGAGCN-MT [19]	SEED; SEED-IV; DREAMER	DE + PSD	Deep GCN	BGAT + Transformer	SD/LOSO	SEED 96.82/89.66
EEG-Conformer [11]	SEED; BCI-IV	Raw EEG	Temporal-spatial CNN	Transformer attention	5-fold CV	SEED 95.30
EEG ST-TCNN [28]	SEED; DEAP	Raw EEG	Parallel Transformers	CNN feature fusion	Independent runs	SEED 96.67
MSDCGTNet [20]	DEAP; SEED; SEED-IV	Raw EEG	Multi-scale dynamic CNN	Gated Transformer + TCN	5-fold CV	SEED 98.85
TDMNN [29]	SEED; SEED-IV; DEAP; DREAMER	4D DE	CNN + LSTM	Temporal-difference MMD	Within-subject 5-fold	SEED 97.20
JAGP [25]	SEED-IV; SEED-V	Pre-extracted EEG	Feature adaptation	Graph label propagation	O2O/M2O	SEED-IV 76.75/78.77
**DSFConformer**	**SEED**	**Raw EEG**	**Multi-scale CNN**	**DSFF + Lite Transformer**	**Block-wise 5-fold**	**96.08**

*Note:* CNN, convolutional neural network; SAE, sparse autoencoder; DNN, deep neural network; SEED, SJTU Emotion EEG Dataset; DEAP, Database for Emotion Analysis using Physiological Signals; MAHNOB-HCI, MAHNOB Human–Computer Interaction database; BiLSTM, bidirectional long short-term memory; BGAGCN-MT, Bridge Graph Attention-based Graph Convolutional Network with Multi-Scale Transformer; EEG, electroencephalography; TDMNN, Temporal-Difference Minimizing Neural Network; JAGP, Joint Feature Adaptation and Graph Adaptive Label Propagation. Performance values are those reported in the original studies under their respective evaluation protocols and are therefore not necessarily directly comparable. Boldface denotes the proposed method and its corresponding experimental setting and result.

**Table 2:** Summary of the SEED Dataset.

Items	Description
Participants	15
Emotion Categories	Positive, Negative, Neutral
Positive Film Clips	Lost in Thailand; Flirting Scholar; Just Another Pandora’s Box
Negative Film Clips	Tangshan Earthquake; Back to 1942
Neutral Film Clips	World Heritage in China
Trial Protocol	Hint of start: 5 s; Movie clip: 240 s; Self-assessment: 45 s; Rest: 15 s

**Table 3:** Hyperparameter Settings Used in the Experiments.

Items	Setting
EEG channels	62
Normalization procedure	In-channel normalization
Batch size	64
Training epochs	100
Early stopping	None
Dropout	0.3 for all layers
Number of branches	3
S-branch kernel size	Kernel size: (1,15); Stride: (1,2); Padding: (0,7)
M-branch kernel size	Kernel size: (1,31); Stride: (1,2); Padding: (0,15)
L-branch kernel size	Kernel size: (1,63); Stride: (1,4); Padding: (0,31)
Embedding dimension	80
Feed-forward dimension	320
Loss function	Cross-Entropy 80% + Focal 20% (γ=2.5); CORAL loss: 20% of total loss
Optimizer	AdamW
Learning rate	0.0005
Lite Transformer layers	8
Attention heads	8
S&R segmentation number	8
Mixup ratio	50% of training data (α=0.2)

**Table 4:** Average Performance on the SEED Dataset.

Methods	Accuracy (%)	Preprocessing	Test Protocol	Source
SVM	86.08	DE	Session-wise	Cited [30]
DGCNN	90.40	DE	Segment-wise	Cited [31]
GCN-LSTM	91.06	DE + PLV	Trial-wise	Cited [32]
GELM	91.07	DE	Segment-wise	Cited [23]
GERBN	92.37	Wavelet + PLV	Trial-wise	Cited [33]
R2G-STNN	93.98	DE	Trial-wise	Cited [6]
ELAI-SGCN	94.63	Raw	Segment-wise	Cited [34]
ATTN-GAN	94.87	DE + PSD	Segment-wise	Cited [35]
EEG-Conformer	95.30	Raw	Segment-wise	Cited [11]
**Proposed**	**96.08**	**Raw**	**Segment-wise**	–

*Note:* Boldface denotes the proposed method and its corresponding results.

**Table 5:** Trial-Wise Comparison Between EEG-Conformer and DSFConformer.

Subject Number	EEG-Conformer (%)	DSFConformer (%)
1	73.12	70.26
2	77.18	80.23
3	74.70	77.44
4	93.99	93.50
5	80.15	80.00
6	65.45	71.11
7	67.22	79.84
8	83.82	70.15
9	81.19	85.60
10	81.04	80.98
11	86.78	84.60
12	78.19	68.73
13	76.28	85.07
14	84.46	88.11
15	77.98	66.50
Average	78.77	78.81

**Table 6:** Trial-Wise Statistical Comparison Between EEG-Conformer and DSFConformer.

Metric	Value
EEG-Conformer mean ± SD	78.77 ± 7.25
DSFConformer mean ± SD	78.81 ± 7.99
Mean difference	+0.04
Median difference	−0.06
95% CI of mean difference	[−3.99, 4.06]
Paired *t*-test	t(14)=0.020,p=0.984
Wilcoxon signed-rank test	W=56.00,p=0.847
Cohen’s $d_{z}$	0.005

**Table 7:** Configurations of Models Used in the Ablation Study.

Model	Multi-Scale Convolution	DSFF	Transformer
Baseline	No	No	Full
M1	Yes	No	Full
M2	Yes	Yes	Full
Proposed	Yes	Yes	Lite

**Table 8:** Statistical Significance Analysis of the Proposed Model Against Ablation Variants.

Compared Models	Δ Acc (%)	*t*-Statistic	*p*-Value	95% CI (%)	Effect Size	Significance
Proposed vs. Baseline	+6.10	8.981	$2.37\times 10^{-7}$	[+4.64,+7.56]	2.319	***
Proposed vs. M1	+0.78	4.017	0.0012	[+0.36,+1.19]	1.037	***
Proposed vs. M2	+0.03	0.207	0.8388	[−0.24,+0.30]	0.053	ns

*Note:* *** indicates statistical significance after Bonferroni correction (p<0.0167); ns indicates no statistically significant difference.

**Table 9:** Computational Efficiency Comparison.

Module	Params	FLOPs	Latency	Convergence Epochs
Standard attention	622,720	2068.48 M	5.38 ms	600
Lite attention	**620,800**	2007.04 M	4.46 ms	50

## Data Availability

Publicly available datasets were analyzed in this study. The SEED dataset can be accessed at https://bcmi.sjtu.edu.cn/home/seed/index.html (accessed on 5 August 2026).
